# Systems serology-based comparison of humoral immune responses induced by liposome or aluminum hydroxide adjuvanted SARS-CoV-2 spike protein

**DOI:** 10.1038/s41598-025-01902-6

**Published:** 2025-05-28

**Authors:** Soo Ji Kim, Taewoo Kim, Satyanarayana Bejjani, Mi Sun Kim, Jung Hyuk Lee, Yuna Shin, Sun-Je Woo, Beom Min Cheon, Doyoung Kim, Sumin Lee, Eunjin Cho, Junhyeon Lee, Ruchirkumar Pansuriya, Wook-Jin Park, Gaurav Pandey, Ravi Ganapathy, Jung-Ah Choi, Ju Yeon Park, Deok Ryun Kim, Cheol-Heui Yun, Jae Seung Yang, Byoung-Shik Shim, Manki Song

**Affiliations:** 1 Science Unit, International Vaccine Institute, Seoul, 08826 Republic of Korea; 2 Department of Agricultural Biotechnology, and Research Institute of Agriculture and Life Sciences, Seoul National University, Seoul, 08826 Republic of Korea; 3 Perelman School of Medicine, Penn Institute for RNA Innovation, University of Pennsylvania, Philadelphia, PA USA

**Keywords:** Adjuvant, Aluminum hydroxide, Liposome, SARS-CoV-2, Systems serology, Adjuvants, Conjugate vaccines, High-throughput screening

## Abstract

Adjuvants play a crucial role in enhancing vaccine-induced immune responses by shaping the magnitude and quality of humoral and cellular immunity. However, the mechanism through which different adjuvants modulate effector functions is not fully understood. Here, we developed an International Vaccine Institute liposome-based adjuvant (ILA) and comprehensively compared humoral immune profiles in mice following the administration of SARS-CoV-2 spike (S) protein formulated with either ILA or aluminum hydroxide (alum) using a systems serology approach. No significant differences were observed in antigen-specific total IgG and neutralizing antibody titers between the two adjuvanted groups. However, the ILA group demonstrated a broader spectrum of humoral immune responses, exhibiting higher levels of antigen-specific IgG2a, IgG2b, and IgG3 compared to the alum group. In addition, S-specific antibody binding to Fcγ receptor (FcγR) 1 and FcγR4 was significantly higher in the ILA group compared to alum. Moreover, Fc-mediated effector functions, such as antibody-mediated monocyte and neutrophil phagocytosis, were significantly more active in the ILA-adjuvanted group. Overall, these findings demonstrate that ILA induces antibodies with superior FcγR binding and Fc-mediated effector functions compared to alum, highlighting its potential role in improving vaccine-induced immunity.

## Introduction

Adjuvants are various molecules that enhance immunogenicity when administered together with vaccine^1^. Aluminum hydroxide (alum) was the only adjuvant approved between the 1920s and 1990s. The oil-in-water emulsion MF59 was approved as an adjuvant for influenza vaccines in 1997^2^. Since then, other adjuvants, such as adjuvant systems (AS) 01, AS03, AS04, and CpG oligodeoxynucleotides (ODN) 1018, have been approved for vaccinations over the past 20 years^3^. This includes liposome-based systems, which are hollow phospholipid bilayer artificial membranes capable of encapsulating and delivering antigens to facilitate their presentation^4^. Liposomes can protect antigens from degradation and prolong their bioavailability, thereby enabling antigen-presenting cells to capture more antigen signals^4^. For instance, Walter Reed Army Institute of Research developed a liposome-based adjuvant known as Army Liposome Formulation (ALF), which contains monophosphoryl lipid A (MPLA) and has been shown to induce higher humoral and cellular immune responses^5^. ALFQ, an adjuvant formulated by adding QS-21 to ALF, further enhances vaccine-induced immunity by promoting Th1 response through MPLA-mediated TLR4 receptor signaling and inducing cross-presentation, and it has demonstrated a promising effect in clinical studies, such as those conducted with a malaria vaccine^6^. Furthermore, AS01, developed by GlaxoSmithKline and approved for human use in the herpes zoster^7^ and malaria vaccines^8^, exerts adjuvant effects similar to ALFQ by inducing MyD88 activation and IFN-γ production through MPLA-mediated TLR4 signaling and by stimulating macrophage secretion of IL-1β and IL-18 via QS-21^9^.

Traditionally, vaccine efficacy has been evaluated based on antigen-specific antibody titers; however, elevated antibody levels do not necessarily equate to effective protection^10^. Identifying the immune correlate of protection which defines the relationship between vaccine-induced immunogenicity and protection is therefore critical for the efficient and accelerated development of vaccines. For example, in the case of pneumococcal vaccines, both antibody titers and opsonophagocytosis are recognized as correlates of protection^11^, while for influenza vaccines, hemagglutination inhibition is considered a key parameter linking immunogenicity to protection^12^. Although neutralizing antibody titers were initially considered the primary correlate of protection for SARS-CoV-2 vaccines^13^, sustained protection against emerging variants of concern has been observed even as these titers wane^14^. The lack of correlation between neutralizing antibody titers from previous vaccination and protection against variants of concern suggests that other factors beyond neutralizing antibody titers may contribute to protection^15,16^. Moreover, in addition to antibody neutralization, non-neutralizing antibodies also play a crucial role in protection against various pathogens, including SARS-CoV-2, through Fc-mediated effector functions, such as antibody-dependent complement deposition (ADCD), antibody-dependent natural killer cell activation, antibody-dependent neutrophil phagocytosis (ADNP), and antibody-dependent cellular phagocytosis (ADCP)^17,18^. To analyze the diverse characteristics and functions of antibodies beyond the measurement of neutralizing titers, the concept of systems serology was introduced^19^. Systems serology comprehensively examines the various attributes of vaccine-induced antibodies and employs statistical analyses to identify features that correlate with protection.

Systems serology is an advanced analytical platform that extends beyond traditional antibody titer measurements to provide a comprehensive assessment of the humoral immune response. This approach integrates high-throughput assays to characterize multiple antibody features, including subclass distribution and Fc-mediated effector functions. By leveraging multivariate statistical analyses, systems serology identifies specific antibody signatures that correlate with protection, offering a more holistic understanding of vaccine-induced immunity^18,20^. Previous studies have utilized systems serology approach to elucidate immune correlates of protection against various pathogens, including SARS-CoV-2. For instance, one study assessing SARS-CoV-2 vaccine responses found that the antibody features, such as ADCP and ADNP, remained associated with protection against infection^21^. Similarly, analyses of convalescent plasma and vaccine-induced immunity have highlighted the role of non-neutralizing antibody functions, including ADCP, ADNP, and complement activation^22^. These findings underscore the necessity of considering a broader range of immune parameters beyond neutralization when defining correlates of protection^23^.

Here, we employed a systems serology approach to compare the humoral immune profiles of alum with the International Vaccine Institute (IVI) liposome-based adjuvant (ILA), a modified version of the ALF adjuvant system. Serum samples from mice that received two doses of SARS-CoV-2 spike (S) protein formulated with either ILA or alum were analyzed for various features, including antibody responses, Fcγ receptor (FcγR) binding profiles, and FcγR-mediated effector functions. While no significant differences in antigen-specific total IgG (tIgG) and neutralizing antibody titers were observed between the two adjuvanted groups, systems serology analysis revealed that ILA induced higher levels of S-specific IgG2a and S-specific antibody binding responses to FcγR1 and FcγR4. In addition, we found that S-specific ADCP and ADNP were significantly higher in the ILA group compared to the alum group. Taken together, these findings suggest that ILA induces more robust functional immune responses compared to the alum. Using this high-throughput systems serology approach, we identified differences that were not observed in conventional antibody titers, such as binding and neutralizing antibodies, highlighting distinct immune features that may guide the optimization of adjuvant strategies and vaccine formulation.

## Results

### Characterization and stability of the ILA

To evaluate the physicochemical properties of the ILA liposomal adjuvant, particle size and composition were assessed. The average particle size of ILA, measured by dynamic light scattering (DLS) using a Zetasizer, was 100 ± 20 d.nm with a polydispersity index of 0.4 ± 0.1 (Supplementary Fig. 1A). Each spectrum (red, green, or black line) represents independent DLS measurements, indicating consistent size distribution. Cryo-transmission electron microscopy (Cryo-TEM) images further confirmed the spherical morphology and uniform distribution of the liposomal particles in PBS buffer (Supplementary Fig. 1B). To analyze the composition of the liposomal components, we conducted quantitative analysis using reverse-phase high-performance liquid chromatography (HPLC) with evaporative light scattering detection (ELSD), which demonstrated the presence of distinct lipid components (Supplementary Fig. 1C). To assess the stability of the ILA formulation, samples were stored at 4 °C for 12 months and evaluated at designated time points. Particle size and lipid composition were monitored using the Zetasizer and HPLC-ELSD, respectively. No significant changes in either size or composition were observed during the 12-month storage period, indicating that the ILA formulation remained physically and chemically stable for at least one year (Supplementary Fig. 1D).

### Antibody responses to the SARS-Cov-2 S protein adjuvanted with ILA or alum

**Fig. 1:**
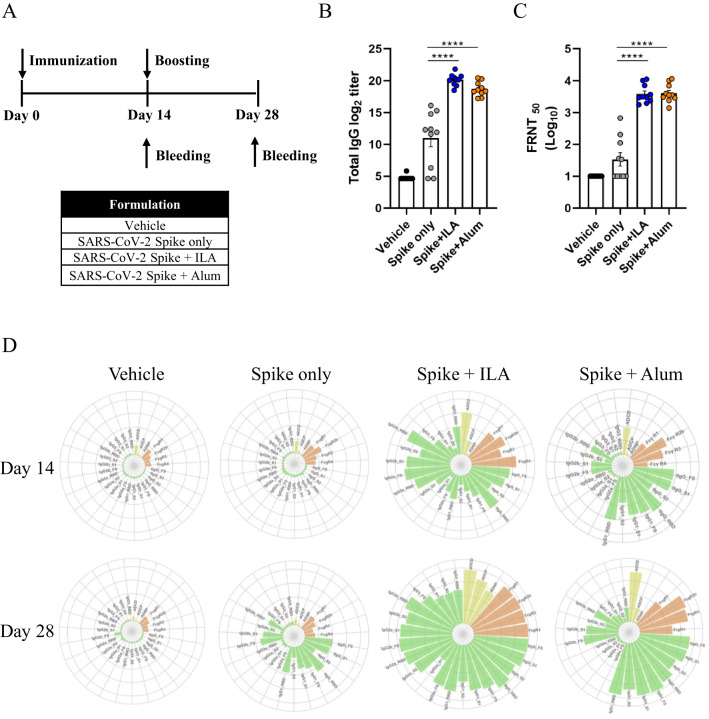
Humoral immune responses induced by the adjuvants. (**A**) schematic overview of the immunization schedule and experimental design. Six- to eight-week-old female K18-hACE2 mice were intramuscularly immunized with 2 μg of recombinant SARS-CoV-2 S protein alone or in combination with adjuvants alum or ILA, in a total volume of 50 μl, on days 0 and 14. PBS, used as a vehicle control, served as the negative control group. (**B**) S protein-binding and (**C**) neutralizing antibody titers in the sera of immunized mice were measured on day 28 after the first immunization via ELISA and FRNT_50_, respectively. Dots represent replicates (n = 10). Tukey’s multiple comparisons test was used following one-way ANOVA. Error bars indicate means ± SEM. *P*-values: *p < 0.05, **p < 0.01, ***p < 0.001, and ****p < 0.0001. (**D**) The circular plots depict the mean percentile of each antibody feature for each adjuvant group at days 14 and 28. Percentile rank scores were determined for each antibody feature across all individuals.

To compare the characteristics of antibodies induced by SARS-CoV-2 S protein formulated with ILA or alum, mice were immunized twice with S protein alone, S protein with ILA, or S protein with alum two weeks apart (Fig. 1A). We then measured antigen-specific binding antibody and neutralizing antibody titers, which are commonly used to assess vaccine immunogenicity. The mice that received two doses of S protein adjuvanted with ILA or alum showed significantly higher levels of S-specific IgG (20.13 ± 0.1 and 18.68 ± 0.13, respectively) compared to those receiving the S protein alone (10.99 ± 0.71), as well as higher neutralizing antibody titers (3.45 ± 1.36 for ILA and 3.43 ± 1.36 for alum), while no significant difference was observed between the ILA and alum groups (Fig. 1B,C).

### Systems serology analysis of humoral immune responses induced by SARS-CoV-2 S formulated with ILA or alum

To further explore how these adjuvants contribute to humoral immune responses, we performed systems serology, an approach that integrates advanced analytical techniques and statistical methods^24^. As shown in Fig. 1D, the S protein alone elicited modest antigen-specific responses after the second immunization, while the S protein with ILA or alum induced a more diverse and higher level of humoral immune responses. Notably, ILA induced a broader spectrum of humoral immune responses compared to alum. In addition, both adjuvant groups showed elevated overall humoral immune responses following the second immunization (Fig. 1D).

### ILA significantly induced robust antigen-specific IgG subclasses

**Fig. 2:**
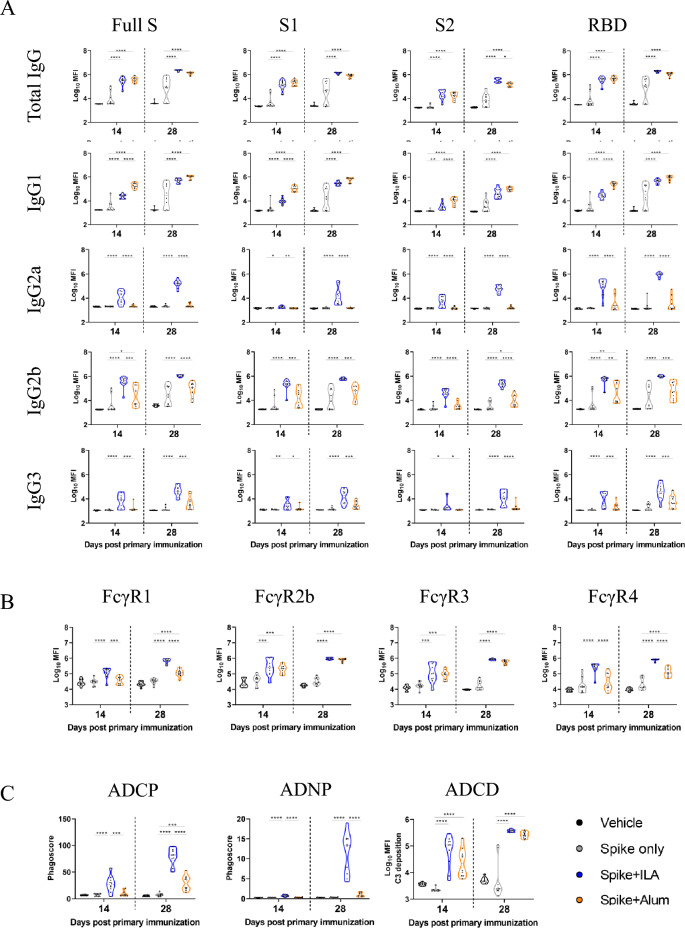
Univariate analysis of functional antibody responses induced by the adjuvants. (**A**) A violin plot showing univariate comparisons of SARS-CoV-2 S, S1, S2, and RBD-specific antibody profiling between the groups. Groups received PBS (used as a vehicle control), S protein alone, or S protein formulated with ILA or alum. Mice were immunized twice at two-week intervals, and sera collected on days 14 and 28 post-initial immunization were used for analysis. Measurements are provided as log_10_ MFI. (**B**) SARS-CoV-2 S-specific FcγR binding was analyzed in day 14 and 28 samples. (**C**) ADCP, ADNP, and ADCD in immunized mice groups. Dots represent replicates (n = 10). The dashed line indicates the median value of each distribution. System serology univariate comparison statistical significance between ILA and alum was analyzed via Mann–Whitney *U* tests. Error bars indicate mean ± SEM. *P* values: *p < 0.05, **p < 0.01, ***p < 0.001, and ****p < 0.0001.

To better understand the specific differences between the two adjuvants, we compared each feature obtained from the multiplexed and quantitative profiling of mouse IgG subclasses against full S, S1, S2, and RBD domains (Fig. 2A). Both adjuvant groups exhibited elevated levels of tIgG and IgG1 against the four different antigens compared to the S protein alone after initial immunization, and these antibody titer differences were sustained following the second immunization. While both adjuvants induced mostly comparable S-specific IgG and IgG1 antibody titers, a significant increase in IgG2a antibody responses against the full S (day 14: 4.06 ± 0.13 for ILA, 3.35 ± 0.03 for alum; day 28: 5.19 ± 0.07 for ILA, 3.40 ± 0.04 for alum), S1 (day 14: 3.28 ± 0.03 for ILA, 3.17 ± 0.01 for alum; day 28: 4.15 ± 0.17 for ILA, 3.19 ± 0.01 for alum), S2 (day 14: 3.70 ± 0.11 for ILA, 3.17 ± 0.03 for alum; day 28: 4.76 ± 0.07 for ILA, 3.21 ± 0.03 for alum), and RBD (day 14: 4.99 ± 0.16 for ILA, 3.47 ± 0.15 for alum; day 28: 5.93 ± 0.04 for ILA, 3.53 ± 0.15 for alum) antigens was observed only in the ILA group after immunization, indicating that ILA induces a stronger Th1-type immune response. Furthermore, the IgG2b and IgG3 titers were both significantly higher in the ILA group compared to the alum group across all antigens after the first immunization, which persisted after the second immunization. These results indicate that while both adjuvants enhanced antigen-specific IgG1 antibody responses, ILA induced a stronger Th1-type immune response than alum.

### ILA exhibited stronger FcγR1- and FcγR4-binding responses than alum

Next, we assessed the profiles of antibodies binding to four FcγRs, which are critical mediators of antibody Fc effector functions (Fig. 2B). Our analysis revealed that both adjuvant groups showed significant increases in S-specific Fc binding to all four FcγRs compared to the S protein alone following the second vaccination. Interestingly, S-specific FcγR1- and FcγR4-binding responses were significantly higher in the ILA group (day 14: 5.06 ± 0.08 and 5.39 ± 0.08, respectively; day 28: 5.83 ± 0.03 and 5.88 ± 0.02, respectively) compared to those in the alum group (day 14: 4.58 ± 0.06 and 4.45 ± 0.31, respectively; day 28: 5.07 ± 0.06 and 5.09 ± 0.07, respectively). This difference was observed after the first immunization and became more distinct after the second immunization. In contrast, antibody binding to FcγR2b and FcγR3 increased to similar levels in both adjuvant groups after the first and second immunizations. Given that mouse IgG2a predominantly binds to FcγR1 and FcγR4^25^, our results suggest that IgG2a in the sera of mice immunized with S with ILA primarily binds to these receptors.

### Functional assays revealed enhanced phagocytosis in the ILA group compared to alum

Previous studies have shown that antibody Fc-mediated effector functions, such as ADCP, ADNP, and ADCD, play a crucial role in protecting against infectious diseases^26^. Therefore, we conducted functional assays using mouse monocytic cells, neutrophil cells, and complements to ascertain the functional properties associated with the antibodies induced by adjuvants (Fig. 2C). The phagocytic scores of monocytes and neutrophils were higher in the ILA group, compared to the other groups after first immunization (ADCP: 2.74 ± 0.48 for ILA and 0.96 ± 0.25 for alum; ADNP: 0.75 ± 0.15 for ILA and 0.34 ± 0.07 for alum), which persisted after the second immunization (ADCP: 8.00 ± 0.30 for ILA and 2.96 ± 0.41 for alum; ADNP: 11.41 ± 0.81 for ILA and 1.00 ± 0.25 for alum). However, both adjuvant groups exhibited higher complement deposition after immunization compared to S protein alone, but no significant differences in ADCD responses were detected between the two adjuvanted groups (day 14: 4.80 ± 0.14 for ILA and 4.37 ± 0.12 for alum; day 28: 5.57 ± 0.01 for ILA and 5.41 ± 0.03 for alum). Taken together, these results indicate that both adjuvants increased complement deposition with no difference in ADCD responses, while ILA significantly enhanced phagocytic responses that may contribute to the protection against SARS-CoV-2 infection.

### Multivariate analysis revealed distinct antibody features across adjuvanted groups

**Fig. 3:**
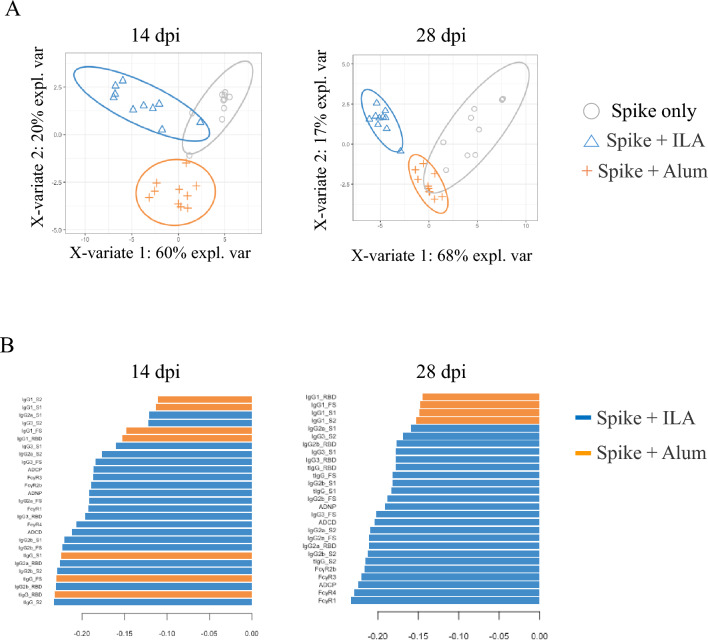
Multivariate analysis of humoral immune responses induced by the adjuvants. (**A**) The PLS-DA model was applied using all antibody features measured from mouse sera collected on days 14 and 28 after the first immunization. Groups included mice immunized with S protein alone, or S protein formulated with ILA or alum (n = 10 per group). (**B**) Loading factors of the first component were generated based on the PLS-DA model. The length and color of the bar represent the contribution level and the group with the highest loading values, respectively.

Next, we employed PLS-DA to identify and visualize the differences in humoral immune profiles elicited by each adjuvant at 14- and 28-days post-immunization (Fig. 3). PLS-DA clearly separated the two adjuvant groups after the first immunization, although the ILA group clusters slightly overlapped with S-only clusters (Fig. 3A). The alum group prominently featured tIgG and IgG1 responses to various domains in the loading plot based on the first principal component after the first immunization. In contrast, most features, such as IgG2b, IgG2a, ADCD, FcγR4, FcγR3, and ADNP, were prominent in the ILA group (Fig. 3B). The separation between the two adjuvanted groups became even more distinct following the second immunization, indicating enhanced immune responses and further differentiation of the immune profiles induced by ILA (Fig. 3A). PLS-DA of component 1 loadings identified features, such as FcγR1, FcγR4, ADCP, FcγR3, FcγR2b, IgG2a, ADCD, IgG3, ADNP, IgG2b, tIgG, and IgG3, as the main ILA group features, whereas only IgG1 was distinguished in the alum group (Fig. 3B). Overall, these results suggest that ILA elicits stronger initial immune responses and drives a more robust and distinct immune profile compared to alum. To this end, sPLS-DA was conducted to identify key features after automatically discarding less important variables through sparsity (Supplementary Fig. 2A). This analysis revealed FcγR1 for full S as the most significant characteristic distinguishing the ILA group loadings of component 1, whereas IgG1 for S1 was the most significant characteristic of alum in component 2 loadings (Supplementary Fig. 2B).

### Correlation network in immune responses induced by adjuvants

**Fig. 4:**
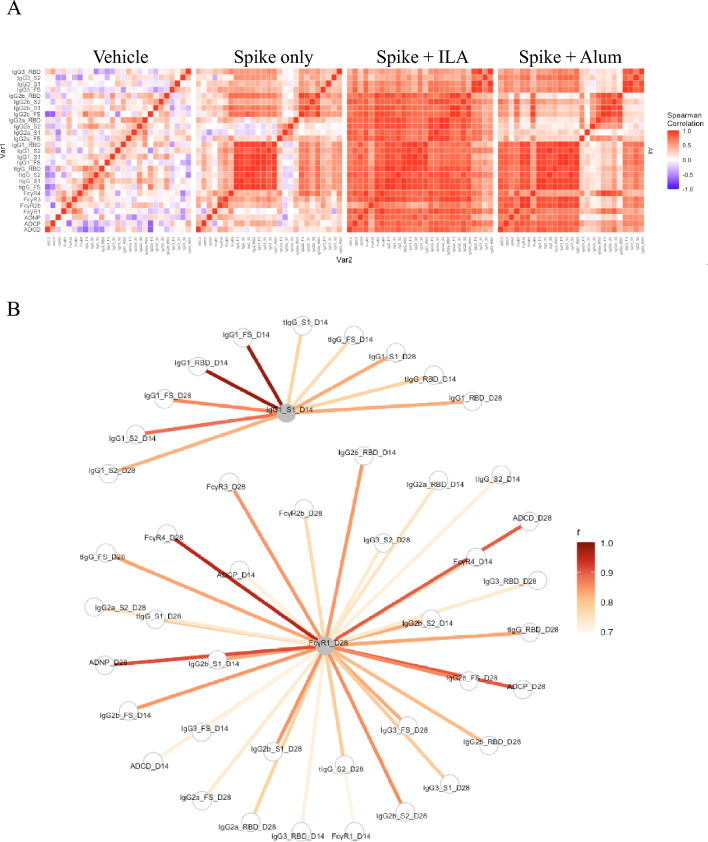
Correlation between immune responses induced by the adjuvants. (**A**) Correlation heatmaps were generated for each group using Spearman rank correlations based on antibody features measured from mouse sera collected on days 14 and 28 after the first immunization. All correlations between humoral features were calculated for each group, with red and blue indicating positive and negative correlations, respectively. (**B**) A correlation network was constructed using selected antibody features derived from day 14 and 28 mouse sera. Spearman’s rank correlation coefficient was calculated.

Finally, the correlation between the overall human immune responses induced by the adjuvants was analyzed to investigate their coordination and strength (Fig. 4A). Notably, ILA showed the most extensive and intense correlations, indicating that it significantly boosted antibody production and FcγR-mediated effector functions. The alum group also exhibited positive correlations, except for IgG2a, IgG2b, and IgG3, although these correlations were not as widespread and intense as those in the ILA group. Furthermore, we analyzed the correlation network of key features that distinguish each adjuvant. We conducted a correlation network analysis to identify additional characteristics associated with key features from sPLS-DA (Fig. 4B). FcγR1 specific to full S was highly correlated with various antibodies and effector functions, such as IgG2b, ADNP, ADCP, and FcγR4. Moreover, IgG1 specific to S1 selected from the alum group correlated with IgG1 and tIgG for various antigens. Overall, these results suggest that ILA significantly enhanced antibody production and Fc receptor-mediated effect functions, with extensive correlations observed across various immune responses.

## Discussion

This study compared the immune responses between liposome and alum adjuvants using systems serology and identified their detailed characteristics, which could not be detected using conventional vaccine evaluation methods such as binding and neutralizing antibody titers. We demonstrated that ILA generated significantly higher antigen-specific IgG2a, IgG2b, and IgG3 responses and S-specific antibody binding to FcγR1 and FcγR4 than alum. IgG2a and IgG2b subclasses have similar functions in complement activation and bind with higher affinity to FcγR1 and FcγR4^27,28^. Notably, IgG2a induces more robust FcγR-mediated activities, enhancing antibody-dependent virus clearance against SARS-CoV-2 infection^29^. In addition, IgG3 antibodies contribute to IgG-mediated effector functions as part of the early immune response, leading to complement activation, triggering inflammatory responses, and facilitating viral neutralization^30^. Moreover, recent studies have identified IgG1, IgG3, and IgM as key protective factors against SARS-CoV-2 infection^31,32^. In light of these results, antigen-specific antibodies binding to FcγR1, FcγR3, and FcγR4 induced by ILA are expected to protect against SARS-CoV-2 infection.

In addition, previous studies have reported that effector functions, such as ADCP, ADNP, and ADCD, contributed to the protection and prevention from severe symptoms of infectious diseases. For example, SARS-CoV-2 convalescent individuals exhibited higher levels of ADCP, ADNP, and ADCD than decedents, suggesting that these markers may correlate with protection^26^. In addition, a mouse study on a SARS-CoV-2 S protein vaccine showed that FcγR4 and ADCP were correlated and contributed to protection^20^. In other mouse studies, the SARS-CoV-2 mRNA vaccine induced high levels of ADCP and ADNP, as well as FcγR3-mediated protection against the Wuhan and Omicron BA.5 strains^17^. Adenovirus vector and subunit vaccine studies determined ADCP and ADNP as correlates of protection against SARS-CoV-2 infection^33^. These studies show that Fc effector functions may play an essential role in protection, which was more strongly exhibited by ILA than alum in this study.

The critical role of FcγR binding in mediating protection has been clearly demonstrated in knockout mouse models. In this study, FcγR knockout mice failed to exhibit protective immunity against SARS-CoV-2 variants following both passive and active immunization, whereas wild-type mice exhibited protection^17^. These findings highlight that FcγR interactions are essential for effective protection. Supporting this, human study have also reported that antibodies capable of engaging FcγRs and eliciting Fc effector functions are associated with increased survival in patients with SARS-CoV-2 infection^34^, further indicating that FcγR engagement is directly linked to protective immunity. Indeed, several studies have demonstrated that enhanced FcγR binding and subsequent effector functions, such as ADCP, are directly associated with improved protective efficacy. For example, FcγR-mediated mechanisms have been shown to contribute to enhanced protection against a variety of viral infections, including SARS-CoV and other pathogens, by facilitating immune cell activation and pathogen clearance^20^.

ILA is a liposome-based adjuvant that contains 3D-PHAD, a synthetic analog of MPLA^35,36^. Its composition is similar to that of the ALF developed by the Walter Reed Army Institute of Research, whose immunological mechanism has been well characterized. Therefore, it is likely that ILA exerts similar immunological actions. The mechanism involves the activation of TLR4 by the adjuvant component 3D-PHAD within the liposome, leading to downstream signaling through MyD88 and NF-κB. This pathway promotes the secretion of pro-inflammatory cytokines such as IL-12 and IL-1β and drives a Th1-biased immune response^3^. The Th1 response in turn promotes the induction of IgG2a, which mediates Fc effector functions critical for protective immunity by binding dominantly to FcγR1 and FcγR4 on macrophages and neutrophils, thereby enhancing ADCP and ADNP activities.

Using systems serology, a clinical study comparing adjuvants in approved hepatitis B vaccines identified key features that distinguished the AS01B, AS01E, and AS03 groups from the AS04 and alum groups^37^. Both univariate and multivariate analyses revealed specific markers distinguishing these groups, including IgG1, FcγR2A, and IgA1. Moreover, all effector functions, such as ADCP, ADNP, and ADCD, were identified as distinct factors of the AS01 and AS03 compared to alum. Importantly, these clinical trial results align with our observation in mice, showing distinctions between the ILA and alum groups. Mouse IgG2a, which corresponds to human IgG1 in clinical studies^38^, was higher in the ILA compared to alum. Our multivariate analysis confirmed that mouse FcγR4, a functional homolog of the human IgG receptor hFcγR3A, was one of the most distinguishing factors between ILA and alum in a human study^39^. Mouse FcγR3, which corresponds to human FcγR2A, showed higher levels in the ILA group compared to the alum group. Furthermore, ADCP and ADNP in the ILA group were also significantly higher than those in the alum group. The only difference between human and mouse studies was the level of IgA, which was not detected in serum under our experimental conditions. The overall parallels between the human and mouse data suggest that the immune responses observed in animal models can serve as reliable predictors of human immunogenicity. This highlights the value of systems serology in animal studies as a powerful tool for predicting vaccine efficacy in humans.

This study has several limitations. While ILA induced a broader immune response than alum, its protective efficacy remains unverified, necessitating further challenge studies. Additionally, as this study only compared alum, future evaluations of other liposome-based adjuvants and vaccine platforms are needed to gain deeper insights into the adjuvanticity of ILA. Lastly, the potential role of T cell responses in protection was not extensively explored and should be addressed in future studies.

This study did not evaluate long-term effects of the vaccine with the adjuvant used; however, existing reports on liposome adjuvants, which examined a long-term vaccine efficacy, provide valuable insights. In a study involving vaccination with the spike protein, antibody levels remained high for up to 180 days after the second immunization, suggesting that the adjuvant effect is sustained over the long term^40^. In this study, ALF, a liposome adjuvant with composition similar to ILA, induced a marked increase in anti-spike IgG and neutralizing antibody responses. Another study on a HIV vaccine using ALF also reported that antibody levels remained elevated for 26 weeks after the first immunization, with a noticeable decline observed only at week 112^41^. In this study as well, ALF elicited strong levels of HIV-specific antibody responses, along with robust ADCP and ADNP activities, consistent with the immune responses induced by ILA. Based on these findings, it is anticipated that ILA will also be capable of inducing durable long-term immunity.

In conclusion, this study demonstrates that ILA, a liposome-based adjuvant, elicits a more robust and diverse humoral immune response than alum, as revealed through systems serology analysis. Notably, ILA enhances antigen-specific IgG subclass responses, FcγR binding, and Fc-mediated effector functions, such as phagocytosis, all of which are critical for protective immunity. These findings underscore the potential of ILA in improving vaccine-induced immunity as a promising alternative to conventional adjuvants.

## Materials and methods

### ILA preparation

ILA was produced at the IVI (Korea). Briefly, ILA was composed of dimyristoylphosphatidylcholine (DMPC) and cholesterol (both in chloroform), dimyristoyl phosphatidylglycerol (DMPG), and monophosphoryl 3-deacyl lipid A (3D-PHAD) (in chloroform: methanol 9:1 v/v)^5,35,42^. All components were obtained from Avanti Polar Lipids (USA) with a purity greater than 99%. Liposomes were prepared through the thin film method via rotary evaporation. Liposome constituents DMPC, DMPG, and 3D-PHAD were dissolved in an organic solvent and placed in a 40 °C water bath until completely dissolved. After mixing each liposome component, the flask was attached to a rotary evaporator and monitored for organic solvent evaporation with a gradual decrease in pressure in thin lipid film formation. The lipid film was further desiccated overnight under vacuum for drying. The lipid film obtained was then rehydrated with Dulbecco’s phosphate-buffered saline (pH 7.2). The lipid film was hydrated with phosphate-buffered saline (PBS, pH 7.2). The lipid film was sonicated, followed by homogenization using Microfluidizer LV1 under high pressure with a cycle time of five to produce multilamellar vesicles into small unilamellar vesicles with a size of 100 ± 20 diameter values in nanometers (d.nm). Final liposomal preparations were subjected to 0.2 µm filtration and were stored at 2 to 8 °C until further use.

### ILA characterization

The liposome compositions (DMPC, DMPG, 3D-PHAD, and cholesterol) were tested and quantified using a reversed-phase high-performance liquid chromatography evaporative light scattering detector (RP-HPLC-ELSD) using Agilent HPLC system as described previously^43^. Briefly, the liposomes were dissolved in methanol at a 1:10 ratio and 100 µL of sample was injected into a C_18_ Column, Luna (Phenomenex) 5 µm 100A, 150 × 4.6 mm with Part No. 00F-4041-E0. The analytes were eluted in a gradient mode using methanol: H_2_O (95:5%) and isopropyl alcohol + 0.1% trifluoroacetic acid as buffers A & B, respectively. ILA size and polydispersity index were measured by the zeta sizer, Malvern. The shape and uni-lamellar structure was confirmed by Cryo-electron microscopy, make Glacios cryoEM performed at Seoul National University.

### Formulation of liposomes and alum

Liposomal formulations were prepared by mixing uni-lamellar liposomes of ILA with 10 µg of 3D-PHAD from a 0.84 mg/mL stock solution. The liposomal formulations were tested before the formulation to confirm the size homogeneity of the ILA for animal experiments. All preparations were vortexed to ensure their homogenous mixing for mice injections. Alum gel (Invivogen, vac-alu-50) was diluted in PBS with Full S protein antigens at a ratio of 1:3 and mixed with a pipette for 5 min to ensure homogeneity.

### Cell culture

Murine monocyte J774A.1 (American Type Culture Collection, ATCC, TIB-67), African green monkey epithelial (Vero) cells (ATCC, CCL-81), and murine lymphoblast 32D clone 3 (ATCC, CRL-3594) were obtained from the ATCC. J774A.1 and Vero cells were cultured at 37 °C and 5% CO_2_ in Dulbecco’s modified Eagle’s medium (DMEM) (Gibco, 11995065) supplemented with 10% heat-inactivated fetal bovine serum (FBS) (Gibco, 26140-079) and 1% penicillin–streptomycin (10,000 U/mL) (Gibco, 15140122). Murine lymphoblast 32D clone 3 cells were cultured in RPMI 1640 medium (Gibco, 11875119) with 10% heat-inactivated FBS, mouse IL-3 (5 ng/mL) (Peprotech, 213-13), and 1% penicillin–streptomycin. The 32D clone 3 cells were differentiated through culture in 10% heat-inactivated FBS, mouse G-CSF (100 ng/mL) (Peprotech, 250-05), and 1% penicillin–streptomycin for seven days before the murine neutrophil phagocytosis assay^44,45^.

### Animal studies

Six to eight-week-old female K18-Tg hACE2 (K18-hACE2) mice (#034860, Jackson Labs) were used for all experiments. All experiments with mice followed protocols approved by the IVI Institutional Animal Care and Use Committee (IACUC, IACUC PN 2023-04) according to the guidelines provided by AAALAC International. All animal reporting was conducted in accordance with ARRIVE guidelines. Mice were randomly assigned to four groups and received two intramuscular immunizations at two-week intervals. The treatment groups included PBS as a control vehicle, S protein (2 μg, AcroBiosystems, SPN-C52H9) alone, or S protein and indicated adjuvants [alum gel (Invivogen, vac-alu) and ILA (10 μg of 3D-PHAD)] with a final injection volume of 50 μL. Each group consisted of 10 animals, and the experiment was performed in duplicate. Prior to immunization and blood collection, mice were anesthetized with 3–5% isoflurane inhalation. Blood samples were collected from the orbital venous sinus two weeks after each immunization. Mice were euthanized using CO_2_ overdose followed by cervical dislocation, in accordance with the AVMA guidelines. All experimental procedures and methods were performed in accordance with applicable regulations and guidelines.

### ELISA

ELISA was performed to measure antibody titers specific to the full SARS-CoV-2 S protein. We coated 96-well flat-bottom Maxisorp microplates (Nunc, 439454) with 70 μL of full S protein (2 μg/mL, AcroBiosystems, SPN-C52H9) in PBS overnight. The plates were washed three times with 0.05% PBS-Tween 20 (PBST) (GenDEPOT, T9100-010) and blocked with blocking buffer (Thermo Fisher Scientific, 37538) for 2 h at room temperature. The plates were washed with PBST three times and incubated with fivefold diluted sera (100 μL) for 2 h at room temperature. Diluted HRP-conjugated anti-mouse tIgG (1:2000) in bovine serum albumin (BSA) (Sigma, A3803-100G) in PBS added to each well, and the sera were washed with PBST three times. Then, TMB microwell peroxidase substrate (SeraCare, 5120-0077) was added for 10 min at room temperature. TMB stop solution (SeraCare, 5150-0021) was added to stop the reaction. The enzymatic activity was measured at 450 nm using a microplate reader (Molecular Devices, Spectramax 340PC384). The antibody titers were determined using SoftMax Pro software (version 7.1.0).

### Focus reduction neutralization test

Vero cells were plated onto 96-well plates (NUNC, 167008) and cultured at 37 °C with 5% CO_2_ for 18 h. A 60 μL mixture containing threefold serially diluted sera (30 μL) and SARS-CoV-2 (30 μL, 180 foci-forming units per well) was incubated at 37 °C and 5% CO_2_ for 30 min. The Vero cells were washed with 200 μL of serum-free DMEM (Invitrogen, 11995065). The virus-sera mixture (50 μL) was added to the Vero cells and incubated at 37 °C for 5 h. The mixture was aspirated post-infection, and the cells were washed with PBS (Gibco, 10010-023). The Vero cells were fixed with 300 μL 10% formalin solution (Sigma, HT501128-4L) at 4 °C overnight. Vero cells were washed with 100 μL PBS after removing formalin, then permeabilized with 100 μL of 100% ice-cold methanol (Sigma, 32213-1L), followed by a 10-min incubation at room temperature. The methanol was then removed, and the cells were washed with 100 μL PBS before blocking with 100 μL blocking buffer [0.5% normal goat serum (Abcam, Ab7481), 0.5% Tween 20 (GenDEPOT, T9100-100) + 5% (w/v) BSA (Sigma, A3803-100G) in PBS] for 30 min at room temperature. The cells were incubated with a 3,000-fold diluted anti-SARS-CoV-2 NP rabbit monoclonal antibody (Sino Biological, 40143-R001) at 37 °C for 1 h. Then, the cells were treated with a 2,000-fold diluted goat anti-rabbit IgG-HRP (Bio-Rad, 170-6515) solution at 37 °C for 1 h after three washes with 200 μL PBS containing 0.1% Tween 20. The cells were washed three times with 200 μl PBS containing 0.1% Tween 20 and once with 200 μL PBS. A 30 μL TrueBlue solution (Seracare, 5510-0030) was added to the Vero cells, and the plates were incubated at room temperature for 30 min. The plates were air-dried after TrueBlue removal, and the foci in each well were counted using a colony reader (Cytation 7, BioTek). The neutralizing antibody titers were determined using SoftMax Pro GxP software (version 7.1.2).

### Fluorescent beads coupled with SARS-CoV-2 S protein

Mag-Avidin microspheres (Luminex, MA-A012-01 for full S protein, MA-A013-01 for S1 protein, MA-A014-01 for S2 protein, and MA-A-15-01 for RBD protein) were purchased for the multiplexed-antigen antibody subclass profiling and FcγR binding assays. The 1.0 μm yellow-green fluorescent beads (Invitrogen, F8776) and 1.0 μm red fluorescent neutravidin beads (Invitrogen, F8775) were used for functional assays. SARS-CoV-2 full S protein (Sino Biological, 40589-V08H4), SARS-CoV-2 S1 protein (Sino Biological, 40591-V08H), SARS-CoV-2 S2 protein (Sino Biological, 40590-V08H), and SARS-CoV-2 RBD protein (Sino Biological, 40592-V08H) were biotinylated (Thermo, A39257) after 2 h of incubation on ice for the multiplex antibody subclass profiling assay. Only the SARS-CoV-2 full S protein was biotinylated for the FcγR binding and functional assays. A total of 1,500,000 Magplex microspheres or 1 μm fluorescent neutravidin beads (1:2 ratio with antigens) were blocked overnight in either isotyping assay buffer (PBS containing 0.1% BSA (Sigma, A3803-100G) and 0.05% Tween-20 (GenDEPOT, T9100-100) for subclass profiling and FcγR binding assay or PBS containing 0.1% BSA for functional assays.

### Antibody subclass and isotype profiling assay

The antibody subclass and isotype-specific to the SARS-CoV-2 S protein were measured^46^. Mag-Avidin microspheres and biotinylated four different antigens (full S, S1, S2, and RBD proteins) were coupled a day before the assay. Antigen-bead complexes were then blocked once with the isotyping blocking buffer (PBS containing 5% BSA) for 30 min at room temperature. Then, 45 μL of antigen-bead complexes in isotyping assay buffer and 5 μL of diluted serum sample in PBS were mixed in each well of a 96-well microplate (NUNC, 163320) and incubated on a 25 × g plate shaker for 2 h at room temperature. Immune complexes were washed three times at the washing station, and 40 μL of diluted PE-conjugated anti-mouse antibodies from Southern Biotech in isotyping assay buffer in different concentrations [tIgG (1:1000, 1030-09), IgG1 (1:200, 1144-09), IgG2a (1:200, 1155-09), IgG2b (1:200, 1186-09), IgG2c (1:1000, 1079-09S), IgG3 (1:200, 1191-09), IgA (1:200, 1165-09)] were added for 1 h at room temperature on a 25 × g plate shaker. Immune complexes were washed twice at the washing station and resuspended with iQue Qsol Buffer (Intellicyt, 91304). A high-throughput flow cytometer (Sartorius, iQues3) acquired the geometric mean fluorescence intensity (gMFI) of each sample. The results are expressed as the average MFI of duplicate tests from each sample.

### FcγR binding assay

The immune complexes of antigens and antibodies to murine FcγRs were quantified^47^. Mag-avidin microspheres and biotinylated full S protein were coupled a day before the assay. Antigen-bead complexes were then blocked once with isotyping blocking buffer (PBS containing 5% BSA) for 30 min at room temperature. Then, 45 μL of antigen-bead complexes in assay buffer and 5 μL of serum sample diluted in PBS were added into each well of 96 well round bottom microplate (NUNC, 163320). The mixture was thoroughly mixed and incubated on a 25 × g plate shaker for two hours at room temperature. Immune complexes underwent three washes at the washing station, followed by the addition of a total volume of 40 μl biotinylated FcRs [FcγR1 (Sino Biological, 50086-M27H-B), FcγR2b (Sino Biological, 50030-M27H-B), FcγR3 (Sino Biological, 50326-M27H-B), FcγR4 (Sino Biological, 50036-M27H-B)] and Streptavidin R Phycoerythrin (Agilent, PJ315) for 1 h at room temperature on a plate shaker at 25 × g. Immune complexes were resuspended with iQue Qsol Buffer (91304, Intellicyt) after three additional washes at the washing stations. The PE MFI for each sample was obtained using a high-throughput flow cytometer (iQue3, Sartorius). The results are presented as the average MFI of duplicate tests for each sample. A high-throughput flow cytometer (iQue3, Sartorius) acquired the gMFI of each sample.

### Murine antibody-dependent monocyte phagocytosis assay

SARS-CoV-2 S protein-specific ADCP was measured^48^. Biotinylated S proteins were coupled with 1.0 μm of yellow-green-fluorescent beads a day before the assay. The beads were washed twice with 5% BSA in PBS on the following day. Then, 10 μL of antigen-bead complexes and 10 μL of diluted mice serum sample were added to each well of a 96-well low attachment round-bottom plate (Costar, 3799) and incubated for 2 h at 37 °C. The immunocomplex was washed once with PBS. Next, 50,000 J774A.1 cells/well in 200 μL of DMEM (Invitrogen, 11995065) with 10% FBS (Gibco, 26140-079) was added to the immune complex, and the plate was incubated for 1 h at 37 °C with 5% CO_2_. The cells were then incubated with ice-cold 5 mM EDTA (Invitrogen, AM9260G) to prevent cell aggregation and fixed with fixation buffer (BD, 554655). The samples were processed through a high-throughput flow cytometer (Sartorius, iQue3). The phagocytic score of each sample was computed using the formula (% of bead-positive cells x gMFI of bead-positive cells/100,000). The results are expressed as the average phagocytic score each sample after duplicate experiments.

### Murine antibody-dependent neutrophil phagocytosis assay

The SARS-CoV-2 S protein-specific ADNP was calculated^49,50^. Biotinylated S proteins were coupled with 1.0 μm yellow-green-fluorescent beads a day before the assay. The beads were blocked overnight in 0.1% PBSA (Sigma, A3803-100G) and washed twice with 5% BSA in PBS. Then, 10 μL of beads and 10 μL of diluted mouse serum sample were added into a 96-well U bottom microplate (NUNC, 163320) and incubated for 2 h at 37 °C and 5% CO_2_. The immune complexes mixed with 50,000 differentiated 32D clone 3 cells/well in 200 μL of 10% FBS (Gibco, 26140-079) RPMI medium (Invitrogen, 11875119) were incubated for 1 h at 37 °C after washing once with PBS. The cells were then stained with CD11b (Biolegend, 101212) and Ly6G (Biolegend, 127628) antibodies (1:250) in PBS for 15 min and fixed with fixation buffer (BD, 554655). The samples were processed through a high throughput flow cytometer (Sartorius, iQues3). The phagocytic scores were calculated using the formula (% of bead-positive cells x gMFI of bead-positive cells/100,000). The results are expressed as the average of duplicate phagocytic scores.

### Antibody-dependent complement deposition assay

The SARS-CoV-2 S protein-specific ADCD was evaluated^51^. Biotinylated S proteins were coupled with 1.0 μm red-fluorescent neutravidin beads in 2:1 ratio a day before the assay. The beads were blocked with PBS with 0.1% BSA (Sigma, A3803-100G) overnight and washed twice with PBS containing 5% BSA. Then, 10 μL of beads and 10 μL of diluted mice serum sample were added into a 96-well U bottom microplate (NUNC, 163320) and incubated for 2 h at 37 °C and 5% CO_2_. Diluted guinea pig complement (1:50) (Cedarlane, CL4051) in RPMI containing 10% heat-inactivated FBS was added to the immune complexes and incubated for 50 min at 37 °C and 5% CO_2_. The complement was washed twice with 15 mM EDTA in PBS, stained with guinea pig complement C3 antibodies (1:100) (MP Biomedicals, 855385) in PBS for 15 min, and fixed with a fixation buffer (BD, 554655). The samples were processed through a high-throughput flow cytometer (Sartorius, iQues3). The complement depositions of immune complexes were measured as the average gMFI of each sample in duplicates.

### Statistical analysis

Statistical significance was determined via one-way ANOVA, followed by Tukey’s multiple-comparisons test when comparing multiple groups in ELISA, FRNT, and univariate analysis. Probability values of *p* < 0.05 were considered significant and denoted using *. Where indicated, *denotes *p* < 0.05, **p < 0.01, ***p < 0.001, and ****p < 0.0001. The error bars in all figures indicate standard error of the mean (SEM). All calculations and visualization were performed with SAS version 9.4, R version 4.3.3, and GraphPad Prism version 10. For statistical analyses, all values were log-transformed (log10), except ADCP and ADNP. A circular plot was constructed to assess and compare the induced immune response over different time points. Each value was normalized by subtracting the minimum value and then dividing by the range, defined as the difference between the maximum and minimum values across all time points; $\frac{{Value_{feature} - Min \left( {All values_{feature} } \right)}}{{Max \left( {All values_{feature} } \right) - Min \left( {All values_{feature} } \right)}}$. Sparse partial least squares discriminant analysis (sPLS-DA) was conducted using the function ‘splsda’ of the R package ‘mixOmics’^52^ to select the important features to discriminate each group. We employed tenfold cross-validation, and the procedure was repeated 10 times to ensure that the most effective combination of components was selected.

## Supplementary Information

Supplementary Information.

## Data Availability

The datasets generated during and/or analyzed during the current study are available from the corresponding author on reasonable request.
