# Supplementary material for: Systems serology-based comparison of humoral immune responses induced by liposome or aluminum hydroxide adjuvanted SARS-CoV-2 spike protein
Source: Sci Rep. 2025 May 28;15:18734. doi: 10.1038/s41598-025-01902-6 (PMC12119814; doi:10.1038/s41598-025-01902-6)
Supplement: Supplementary file 1 — Supplementary Information. [file 41598_2025_1902_MOESM1_ESM.pdf]

Systems Serology-Based Comparison of Humoral Immune Responses Induced by Liposome or Aluminum Hydroxide Adjuvanted SARS-CoV-2 Spike Protein

Soo Ji Kim[1,2], Taewoo Kim[1], Satyanarayana Bejjani[1], Mi Sun Kim[1], Jung Hyuk Lee[1], Yuna Shin[1], Sun-Je Woo[1], Beom Min Cheon[1], Doyoung Kim[1,2], Sumin Lee[1], Eunjin Cho[1], Junhyeon Lee[1], Ruchirkumar Pansuriya[1], Wook-Jin Park[3], Gaurav Pandey[1], Ravi Ganapathy[1], Jung-Ah Choi[1], Ju Yeon Park[1], Deok Ryun Kim[1], Cheol-Heui Yun[2], Jae Seung Yang[1,*], Byoung Shik Shim[1,*], Manki Song[1,*]

[1]Science unit, International Vaccine Institute, Seoul, 08826, Republic of Korea
[2]Department of Agricultural Biotechnology, and Research Institute of Agriculture and Life Sciences, Seoul National University, Seoul, 08826, Republic of Korea
[3]Penn Institute for RNA Innovation, Perelman School of Medicine, University of Pennsylvania, Philadelphia, PA, USA

*Address correspondence to Jae Seung Yang, jsyang@ivi.int; Byoung-Shik Shim, byoungshik.shim@ivi.int; Manki Song, mksong@ivi.int

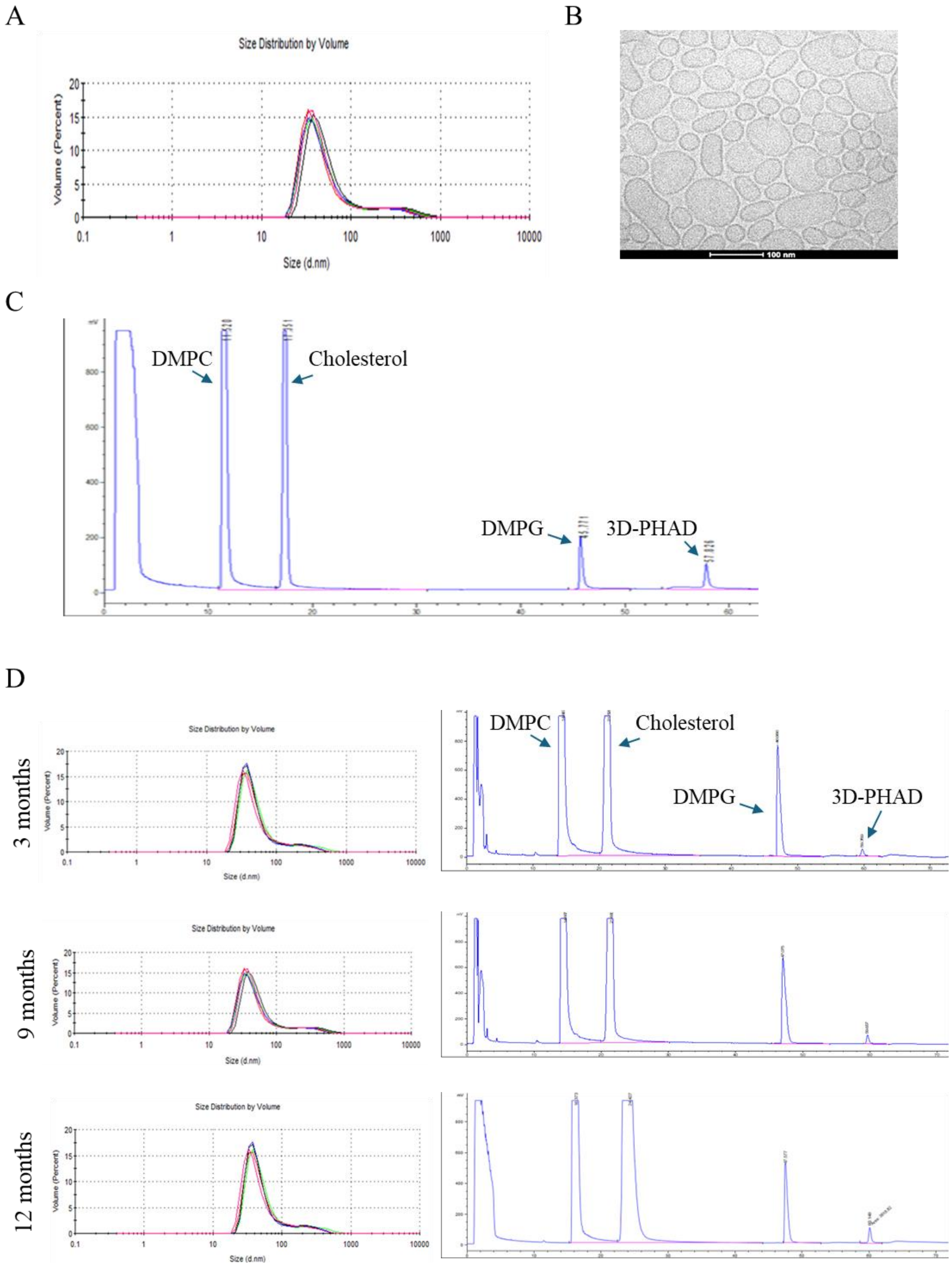
A
Size Distribution by Volume
Volume (Percent)
Size (d.nm)
B
100 nm
C
DMPC
Cholesterol
DMPG
3D-PHAD
D
3 months
9 months
12 months
Size Distribution by Volume
Volume (Percent)
Size (d.nm)
DMPC
Cholesterol
DMPG
3D-PHAD

Supplementary Figure 1. Characterization and stability of ILA

(A) Particle size of ILA measured using a Zetasizer. Y-axis indicates volume percent of scattered light; X-axis shows particle diameter in nanometers. Red, green, and black lines represent independent DLS spectral readings. (B) Cryo-TEM images showing the morphology of ILA in PBS buffer. The bar scale represents 100 nm. (C) Quantitative analysis of lipid components using reverse-phase HPLC with ELSD detection. Y-axis shows detector response in mV; X-axis indicates retention time in minutes. (D) Stability assessment of ILA after storage at 4°C for 12 months.

A

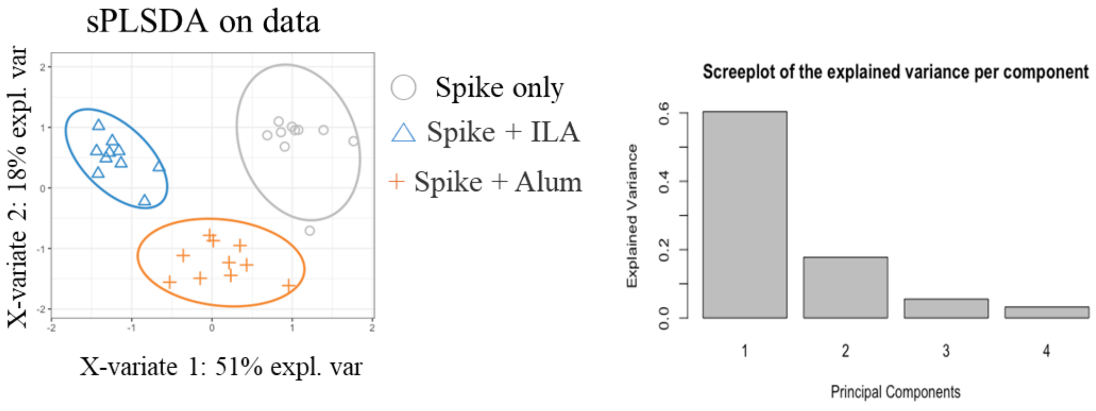
sPLSDA on data
X-variate 2: 18% expl. var
X-variate 1: 51% expl. var
Spike only
Spike + ILA
Spike + Alum
Screeplot of the explained variance per component
Explained Variance
0.0
0.2
0.4
0.6
1
2
3
4
Principal Components

B

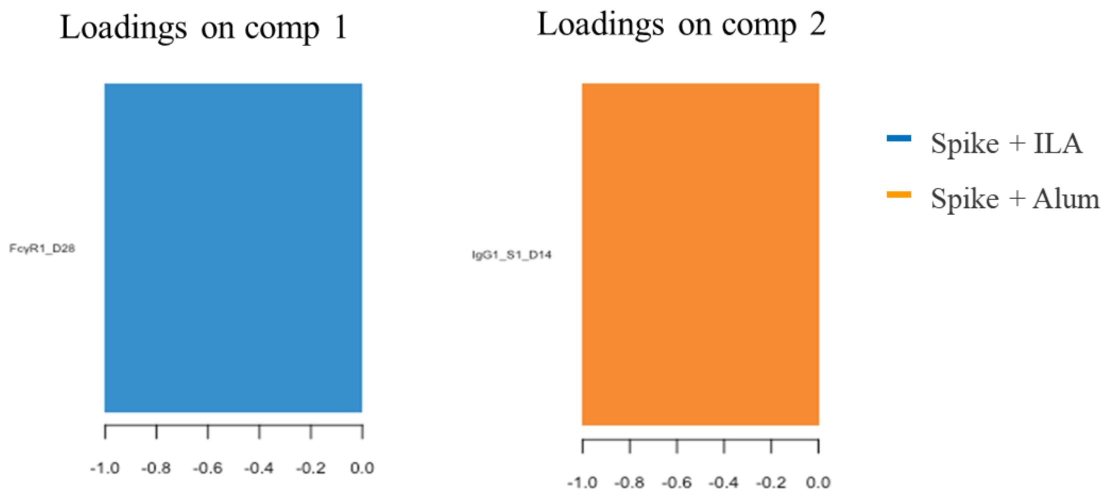
Loadings on comp 1
FcγR1_D28
-1.0
-0.8
-0.6
-0.4
-0.2
0.0
Loadings on comp 2
IgG1_S1_D14
-1.0
-0.8
-0.6
-0.4
-0.2
0.0
Spike + ILA
Spike + Alum

Supplementary Figure 2. sPLSDA of humoral immune responses

(A) The sPLS-DA model was applied with all features. (B) The loading factors of the first component were generated based on the sPLS-DA model. The length and color of the bar represent the contribution level and the group with the highest loading values, respectively.
